# The efficacy of nitroglycerin to prevent radial artery spasm and occlusion during and after transradial catheterization: A systematic review and meta‐analysis of randomized controlled trials

**DOI:** 10.1002/clc.23906

**Published:** 2022-11-06

**Authors:** Basel Abdelazeem, Mohamed T. Abuelazm, Sarya Swed, Mohamed Gamal, Mostafa Atef, Mohamed A. Al‐Zeftawy, Muhammad A. Noori, Anthony Lutz, Annabelle S. Volgman

**Affiliations:** ^1^ McLaren Health Care Flint Michigan USA; ^2^ Michigan State University East Lansing Michigan USA; ^3^ Faculty of Medicine Tanta University Tanta Egypt; ^4^ Faculty of Medicine Aleppo University Aleppo Syria; ^5^ Faculty of Medicine Cairo University Cairo Egypt; ^6^ Rutgers Health/Trinitas New Jersey USA; ^7^ Division of Cardiology Beaumont Hospital Farmington Hills Michigan USA; ^8^ Division of Cardiology Rush University Medical Center Chicago Illinois USA

**Keywords:** cardiac intervention, nitroglycerin, radial artery occlusion, radial artery spasm, RAO, RAS, transradial

## Abstract

Radial artery spasm (RAS) is the most common cause of transradial access site crossover and is a common intra‐procedural complication. RAS incidence can lead to radial artery occlusion (RAO) postprocedure, preventing the radial artery as a future access site. We evaluated the efficacy of nitroglycerin preventing RAS and RAO during transradial catheterization discussing the different routes of administration, including topical, subcutaneous, and intra‐arterial. A systematic review and meta‐analysis included all relevant articles until April 23, 2022. We searched six databases Google Scholar, Web of Science, SCOPUS, EMBASE, PubMed (MEDLINE), and CENTRAL. We registered our review protocol in PROSPERO with ID: CRD42022330356. We included 11 trials with 5814 patients. Compared to placebo, the pooled analysis favored subcutaneous nitroglycerin in preventing RAS (risk ratio [RR]: 0.57 with 95% confidence interval [CI] [0.43–0.77], *p* = .0003) and RAO (RR: 0.39 with 95% CI [0.16–0.98], *p* = .05). In contrast to the intra‐arterial nitroglycerin that showed nonstatistically significant results in preventing RAS and RAO (RR: 0.8 with 95% CI [0.63–1.02], *p* = .07)‐ (RR: 0.78 with 95% CI [0.6–1.01], *p* = .06)), respectively. Also, topical nitroglycerin did not prevent RAS (RR: 0.73 with 95% CI [0.42–1.24], *p* = .24). Compared with placebo, subcutaneous nitroglycerin during transradial catheterization reduced the incidence of RAS and RAO. Meanwhile, Intra‐arterial and topical nitroglycerin did not show statistically significant outcomes. Subcutaneous nitroglycerin may be a practical and cost‐effective technique to facilitate transradial catheterization; however, more RCTs are needed to evaluate the subcutaneous versus intra‐arterial nitroglycerin administration.

## 1 INTRODUCTION

The transradial artery access for coronary angiography was first reported in 1989.^1^ In most countries, the transradial artery (TRA) has become the most common access point for cardiac catheterization, with a class 1 indication in the 2017 European Society of Cardiology guidelines on the management of patients with acute coronary syndrome.^2^ Using the TRA approach has grown in popularity because of the lower risk of bleeding and vascular complications compared to trans‐femoral access.^3^, ^4^, ^5^ Moreover, TRA is safe and cost‐effective, with an equivalent procedural success rate compared with the femoral approach.^6^ Although the use of TRA is growing, radial artery spasm (RAS) and radial artery occlusion (RAO) continue to be one of the limitations of transradial access, ranging from 5% to 30% and 1% to 10%, respectively.^7^, ^8^, ^9^

RAS is the sudden and temporary narrowing of the radial artery.^10^ Furthermore, RAS is a common intra‐procedural complication and the most frequent cause of transradial access site crossover. RAS is detected by various clinical criteria, such as limitation in catheter mobility, pain reported by the patient, resistance in catheter maneuvring, or difficulty in removing the catheter.^11^, ^12^ Female gender, low BMI, younger age, small radial artery diameter, large sheath, artery size ratio, and multiple catheter exchanges are the predictors of RAS.^11^ Operator experience is also a significant factor that can influence the incidence of RAS, leading to incidence rate variations across centers with different levels of experience.^13^ While RAO results from thrombus formation due to intimal injury during catheterization. RAO may cause potential discomfort and eventually lead to procedure failure. It can also limit the radial artery's usability as an access site in the future.^14^ RAO can be identified by a visible obstruction on the two‐dimensional ultrasound or by the absence of a Doppler flow signal distal to the puncture site.^13^

To reduce the RAO incidence, various strategies have been utilized, including using a small‐caliber catheter, anticoagulation with heparin, maintenance of patency during hemostasis, and short‐term compressive dressing.^15^ Vasodilators like nitroglycerin and verapamil have also been used to reduce RAO risk. Using nitroglycerin (a nitric oxide donor) at the end of a TRA procedure is believed to reduce the risk of RAO by reducing the inflammation and intimal hyperplasia in the radial artery, which is subjected to local trauma by TRA.^16^ Facilitation of radial artery access via radial artery dilatation with the use of topical, sublingual, and subcutaneous nitroglycerin also reduces the risk of RAS by minimizing the number of puncture attempts.^17^, ^18^, ^19^

Previous randomized controlled trials (RCTs) have evaluated the different routes of nitroglycerin administration: topical,^20^, ^21^ subcutaneous,^17^, ^22^, ^23^, ^24^, ^25^ and intra‐arterial^8^, ^13^, ^26^, ^27^ to prevent RAS and RAO. This systemic review and meta‐analysis aims to evaluate the efficacy of nitroglycerin in preventing RAS and RAO during transradial catheterization and to discuss the different routes of nitroglycerin administration.

## 2 METHODOLOGY

### 2.1 Protocol registration

For this systematic review and meta‐analysis, we strictly followed the Preferred Reporting Items for Systematic Reviews and Meta‐Analyses (PRISMA) statement^28^ and the Cochrane Handbook of Systematic reviews and meta‐analysis.^29^ The PRISMA 2020 checklist is clarified in Table S1. We registered and published our review protocol in PROSPERO with ID: CRD42022330356.

### 2.2 Data sources and search strategy

Two reviewers (B.A. and M.G.) systematically searched the following electronic databases: Google Scholar, Web of Science, SCOPUS, EMBASE, PubMed (MEDLINE), and Cochrane Central Register of Controlled Trials until 23 April 2022. We used the following search terms: “nitroglycerin,” “vasodilators,” “radial artery spasm,” “radial artery occlusion,” and “transradial.” We did not use any search filters. The detailed search strategy and results are demonstrated in Table S2.

### 2.3 Eligibility criteria

We included RCTs with the following PICO criteria: population (P): adult patients undergoing cardiac diagnostic or intervention transradial catheterization; intervention (I): nitroglycerin regardless of dosage and route of administration; control (C): placebo; outcomes (O): primary outcomes: incidence of RAS detected by various clinical criteria (limitation in catheter mobility, pain reported by patient, resistance in catheter maneuvering, or difficulty in removing the catheter) during or pre‐catheterization, and incidence of RAO detected by the absence of anterograde flow detected by vascular duplex Doppler ultrasound following catheterization for up to 24 h. Our secondary outcomes are radial artery diameter, procedure time, radial artery puncture attempts, and incidence of adverse events (hematoma, residual arm pain, hand ischemia, hypotension, and headache).

We excluded animal studies, pilot studies, observational studies (cohort, case–control, cross‐sectional, case series, and case reports), single‐arm clinical trials, in vitro studies (tissue and culture studies), book chapters, editorials, press articles, and conference abstracts.

### 2.4 Study selection

Using Covidence,^30^ two reviewers (M.G. and S.S.) independently screened the titles and abstracts of the included records after duplicates were removed by Covidence online software.^30^ Then they screened the full texts of the relevant records for the previous eligibility criteria. A third reviewer (M.A.A.) was invited to resolve any conflict.

### 2.5 Data extraction

Using a pre‐tested extraction sheet, four reviewers (M.A., M.A.A., M.G., and S.S.) independently extracted the following data from the included articles: study characteristics (first author name, year of publication, country, study design, total participants, catheterization indication, nitroglycerin's dose, route of administration, and time of administration; any adjuvant drug's dose, route of administration, and time of administration; method of radial artery occlusion assessment); baseline information (age, sex, height, weight, basal metabolic index, smoking, hypertension, diabetes, dyslipidemia, and acute coronary syndrome); efficacy outcomes data (incidence of RAS, RAO, radial artery diameter, procedure time, and radial artery puncture attempts); safety outcomes data (hematoma, residual arm pain, hand ischemia, hypotension, and headache). Conflicts were resolved through discussion.

### 2.6 Risk of bias and quality assessment

Four reviewers (M.A., M.A.A., M.G., and S.S.) independently assessed the included studies for the risk of bias (ROB) using The Cochrane Collaboration's tool for assessing the risk of bias in randomized trials,^31^ based on the following domains: random sequence generation (selection bias), allocation concealment (selection bias), blinding of participants and personnel (performance bias), blinding of outcome assessment (detection bias), incomplete outcome data (attrition bias), selective reporting (reporting bias), and other potential sources of bias. Conflicts were resolved by discussion. Two reviewers (M.A.A. and M.G.) used the Grading of Recommendations Assessment, Development and Evaluation (GRADE) Working Group recommendation^32^, ^33^ for quality of evidence assessment. We considered inconsistency, imprecision, indirectness, publication bias, and risk of bias. Our conclusions on the quality of evidence were justified, recorded, and included in the results reporting for each outcome. A third reviewer (M.T.) resolved any conflicts.

### 2.7 Statistical analysis

We conducted the statistical analysis using RevMan v5.3 software.^34^ We pooled the continuous outcomes using mean difference (MD) with a 95% confidence interval (CI), and we pooled dichotomous outcomes using risk ratio (RR) with 95% (CI). We evaluated heterogeneity using *I* ^2^ and Chi‐square tests; the Chi‐square test assesses whether there is significant heterogeneity, while the I‐square evaluates the magnitude of heterogeneity. According to the Cochrane Handbook (Chapter 9),^29^ an α level below .1 is considered to be a significant heterogeneity (for the Chi‐square test), and the *I* ^2^ test is interpreted as follows: (0%–40%: might not be important; 30%–60%: may represent moderate heterogeneity; 50%–90%: may represent significant heterogeneity). With significant heterogeneity, we used the random‐effects model; otherwise, we used the fixed‐effects model.

In the case of significant heterogeneity, we performed a sensitivity analysis by removing one study at a time and repeating the analysis to analyze the influence of each study on the overall effect size of the outcomes. Moreover, we performed a subgroup analysis based on the route of nitroglycerin administration to test the stability of our results. We did not present funnel plots to identify publication bias because we only included less than 10 studies in each outcome, as recommended by Egger et al.^35^

## 3 RESULTS

### 3.1 Search results and study selection

Two thousand six hundred ninety‐two records were retrieved after the searching process. Eight hundred fourteen duplicates were removed using Covidence. We screened 1878 titles and abstracts, excluding 1841 irrelevant records, then we screened 37 full‐text articles, and finally included 11 articles in our systematic review and meta‐analysis. The selection process is demonstrated in a PRISMA flow chart (Figure S1).

### 3.2 Characteristics of included studies

We included 11 trials^8^, ^13^, ^17^, ^20^, ^21^, ^22^, ^23^, ^24^, ^25^, ^26^, ^27^ with a total of 5814 patients; 2938 in the nitroglycerin group and 2876 in the placebo group. The frequency and percentage of comorbidities, including smoking, hypertension, diabetes, dyslipidemia, and acute coronary syndrome are demonstrated in Table 1. Nitroglycerin was administrated by three routes: subcutaneous,^17^, ^22^, ^23^, ^24^, ^25^ topical,^20^, ^21^ and intra‐arterial.^8^, ^13^, ^26^, ^27^ Procedure characteristics of the included studies, including nitroglycerin and adjuvant drugs, are demonstrated in Table 2. RAS incidence was reported in eight studies,^8^, ^13^, ^17^, ^20^, ^21^, ^22^, ^23^, ^25^ while RAO incidence was reported in four studies.^13^, ^23^, ^24^, ^26^ Furthermore, the safety was estimated by documenting the post manifestations of nitroglycerin administration, including hematoma, hypotension, and headache.

**Table 1:** Baseline characteristics of the included studies.

Study	Number		Age, mean (SD)		Female, N (%)		BMI, mean (SD)	
	Placebo	NG	Placebo	NG	Placebo	NG	Placebo	NG
**Chen et al. 2006** ^8^	93	133	64.2 (11.7)	63.5 (10.3)	30 (32)	46 (41)	N/A	N/A
**Dharma et al. 2014** ^26^	853	853	59.38 (10.23)	59.15 (10.53)	275 (32)	264 (31)	26 (5‐50)	26 (15‐45)
**Da Silva et al. 2022** ^13^	1020	1020	62.06 (10.29)	61.52 (10.35)	399 (39.1)	375 (36.8)	28.51 (7.48)	28.67 (7.67)
**Da Silva et al. 2019** ^27^	164	164	59.79 (11.15)	60.93 (11.9)	58 (35)	58 (35)	28.13 (7.33)	28.47 (7.45)
**Candemir et al. 2009** ^23^	30	33	60.1 (8.2)	61.4 (8.8	4 (13)	3 (9)	N/A	N/A
**Ezhumalai et al. 2014** ^17^	100	100	55.1 (9.6)	53.1 (9.1)	42 (42)	39 (39)	N/A	N/A
**Kiani et al. 2017** ^22^	73	71	56.2 (10.9)	55.1 (9.5)	13 (17.8)	13 (18.3)	28.1 (15.5)	27.3 (4.4)
**Chen et al. 2018** ^24^	94	94	62.9 (9.1)	61.5 (8.4)	34 (36.1)	35 (37.2)	N/A	N/A
**Coroleu et al. 2021** ^25^	357	379	65.1 (10.1)	64.9 (10.1)	137 (38.7)	149 (39.3)	28.4 (4.2)	28.5 (4.2)
**Beyer et al. 2013** ^20^	40	43	59.0 (12.8)	63.4 (12.6)	14 (35)	15 (34)	28.7 (6.0)	28.0 (5.6)
**Gopalkrishnan et al. 2020** ^21^	52	48	66.4 (9.2)	67.4 (9.8)	16 (30)	21 (44)	N/A	N/A

Study	Comorbidities, N (%)										Indication, N (%)			
	Smoking		Hypertension		Diabetes		Dyslipidemia		Acute coronary syndrome		Coronary angiography		PCI	
	Placebo	NG	Placebo	NG	Placebo	NG	Placebo	NG	Placebo	NG	Placebo	NG	Placebo	NG
**Chen et al. 2006** ^8^	48 (51.9)	66 (48.7)	N/A	N/A	62 (67)	93 (68.7)	N/A	N/A	N/A	N/A	N/A	N/A	N/A	N/A
**Dharma et al. 2014** ^26^	N/A	N/A	N/A	N/A	201 (23)	212 (25)	N/A	N/A	245 (29)	241 (28)	510 (59)	515 (60)	340 (39)	337 (39)
**Da Silva et al. 2022** ^13^	226 (22.2)	228 (22.4)	757 (74.2)	775 (76)	368 (36.1)	379 (37.2)	661 (64.8)	612 (60)	478 (46.9)	483 (47.4)	N/A	N/A	N/A	N/A
**Da Silva et al. 2019** ^27^	N/A	N/A	N/A	N/A	N/A	N/A	N/A	N/A	N/A	N/A	N/A	N/A	N/A	N/A
**Candemir et al. 2009** ^23^	16 (53)	19 (58)	23 (77)	21 (64)	11 (33)	8 (24)	7 (23)	13 (39)	N/A	N/A	N/A	N/A	N/A	N/A
**Ezhumalai et al. 2014** ^17^	32 (32)	36 (36)	49 (49)	43 (43)	63 (63)	58 (58)	70 (70)	78 (78)	N/A	N/A	N/A	N/A	N/A	N/A
**Kiani et al. 2017** ^22^	23 (31.5)	24 (33.8)	N/A	N/A	15 (20.5)	11 (15.5)	N/A	N/A	N/A	N/A	N/A	N/A	N/A	N/A
**Chen et al. 2018** ^24^	37 (41.1)	37 (40.7)	54 (60.0)	60 (65.2)	25 (27.8)	30 (30.2)	N/A	N/A	N/A	N/A	61 (67.8%)	58 (63.0%)	29 (32.2%)	34 (37.0%)
**Coroleu et al. 2021** ^25^	139 (38.9)	153 (40.3)	243 (68.1)	277 (73.1)	71 (19.9)	97 (25.6)	176 (49.3)	189 (49.8%)	173	167	248 (69.5%)	259 (68.3%)	9 (2.5%)	15 (3.9%)
**Beyer et al. 2013** ^20^	23 (57.5)	12 (27.9)	N/A	N/A	21 (52.5)	26 (60.4)	N/A	N/A	11 (26)	6 (14)	N/A	N/A	15 (36)	13 (33)
**Gopalkrishnan et al. 2020** ^21^	N/A	N/A	N/A	N/A	N/A	N/A	N/A	N/A	N/A	N/A	N/A	N/A	N/A	N/A

Abbreviations: BMI, basal metabolic rate; SD, standard deviation; N/A, not available; NG, nitroglycerin; PCI, percutaneous coronary intervention.

**Table 2:** Summary characteristics of the included studies

Study	Country	Total Patients	Nitroglycerin			Adjuvant drugs		Sheath size	Methods of RAS or RAO detection	Time of RAS or RAO detection
			Route	Dose (volume)	Time of administration	Before sheath insertion	After sheath insertion			
Chen et al. 2006^8^	Taiwan	226	Intra‐ arterial	100 μg	N/A	2% Xylocaine	Heparin 3000 u (via sheath)	10‐cm‐long, 6‐french Terumo hydrophilic sheath.	Patients’ feeling of pain or withdrawing the catheters or guide wires	During procedure
Dharma et al. 2014^26^	Indonesia, India, and Macedonia	1706	Intra‐ arterial	500 μg	After sheath insertion at the end of the procedure	N/A	Heparin 50–100u (IV)	5‐6‐7‐8 French sheaths	High‐resolution ultrasound or Color Duplex ultrasound studies	24 h following procedure
Da Silva et al. 2022^13^	Brazil	2040	Intra‐ arterial	500 μg	After sheath insertion before or at the end of the procedure.	1% lidocaine	Heparin 5000u (via sheath)	5‐ or 6‐F hydrophilic‐coated short sheath	Pain in the forearm confirmed by DUS	24 h and 30 days after procedure
Da Silva et al. 2019^27^	Brazil	328	intra ‐arterial	200 μg	After sheath insertion	N/A	N/A	N/A	Pain scale	N/A
Candemir et al. 2009^23^	Turkey	63	SC	500 μg (0.1 ml)	Before percutaneous procedures	Prilocaine 2% 18 mg	Verapamil 2.5 mg, heparin 5000u (via sheath)	12‐cm 6‐F hydrophilic sheath	Ultrasonography	N/A
Ezhumalai et al. 2014^17^	India	200	SC	500 μg (1 ml)	Before percutaneous procedures	Lignocaine 2% 2 ml	N/A	N/A	Ultrasonography	Pre‐cannulation
Kiani et al. 2017^22^	Iran	144	SC	500 μg	N/A	Lidocaine hydrochloride 2% 10 mg	N/A	N/A	Severe and painful limitation in the maneuvering of the catheter and the flattening of the arterial waves	N/A
Chen et al. 2018^24^	China	188	SC	500 μg (0.5 ml)	Before percutaneous procedures	2% Lidocaine	Heparin 3000 u (via sheath)	6F hydrophilic sheath	Visible obstruction on 2‐dimensional ultrasound and the absence of Doppler flow signal	24 h following procedure
Coroleu et al. 2021^25^	Argentina	736	SC	200 μg(2 ml)	Before percutaneous procedures	N/A	Heparin 5000u (bolus injection)	N/A	N/A	N/A
Beyer et al. 2013^20^	USA	83	Topical	30 mg ointment	Before percutaneous procedures	Lidocaine cream 40 mg	Heparin 50–70 u/kg (IV)	at baseline 6 French sheath After dilatation, 7 French sheath	Resistance to advancing the catheter through the radial artery, by difficulty in torquing the catheter, or by difficulty in removing the catheter	N/A
Gopalkrishnan et al. 2020^21^	USA	100	Topical	30 mg	Before percutaneous procedures	Lidocaine cream 40 mg	N/A	N/A	Radial artery spasm score	N/A

Abbreviations: mg, milli‐gram; ml, milliliter; μg, micro‐gram; N/A, not available; RAS, radial artery spasm; RAO, radial artery occlusion; SC, subcutaneous; u, unit.

### 3.3 Risk of bias and quality of evidence

According to Cochrane's risk of bias tool, the quality of the included trials ranged from moderate to high. We demonstrated the summary of the risk of bias in Figure S2. Furthermore, our justifications for unclear and high risk of bias assessment are demonstrated in Table S3. The quality of evidence using The GRADE system is illustrated in Table 3.

**Table 3:** GRADE evidence profile

Certainty assessment							№ of patients		Effect		Certainty	Importance
No of studies	Study design	Risk of bias	Inconsistency	Indirectness	Imprecision	Other considerations	Nitroglycerin	Placebo	Relative (95% CI)	Absolute (95% CI)		
**Radial artery spasm**												
8	Randomized trials	Not serious	Not serious	Not serious	Serious^a^	None	190/1829 (10.4%)	258/1765 (14.6%)	**RR 0.71** (0.60–0.85)	**42 fewer per 1000** (from 58 fewer to 22 fewer)	⊕⊕⊕◯ Moderate	CRITICAL
**Radial artery occlusion**												
4	Randomized trials	Serious^b^	Not serious	Not serious	Serious^a^	None	102/1996 (5.1%)	138/1993 (6.9%)	**RR 0.74** (0.58–0.94)	**18 fewer per 1000** (from 29 fewer to 4 fewer)	⊕⊕◯◯ Low	CRITICAL
**Radial artery diameter**												
5	Randomized trials	Serious^b^	Very serious^c^	Not serious	Serious^d^	None	1455	1430	‐	MD **0.27 higher** (0.06 higher to 0.47 higher)	⊕◯◯◯ Very low	CRITICAL
**Procedure duration in minutes**												
6	Randomized trials	Serious^b^	Not serious	Not serious	Very serious^e^	None	2539	2514	‐	MD **0.05 lower** (0.41 lower to 0.3 higher)	⊕◯◯◯ Very low	CRITICAL
**Radial artery puncture attempts**												
5	Randomized trials	Not serious	Very serious^c^	Not serious	Very serious^e^	None	1603	1580	‐	MD **0.21 lower** (0.44 lower to 0.02 higher)	⊕◯◯◯ Very low	CRITICAL
**Adverse events**												
7	Randomized trials	Not serious	Not serious	Not serious	Very serious^f^	None	86/2632 (3.3%)	63/2574 (2.4%)	**RR 1.32** (0.96–1.80)	**8 more per 1000** (from 1 fewer to 20 more)	⊕⊕◯◯ Low	IMPORTANT

Explanations:

^a^ The 95% confidence interval (CI) does not exclude the risk ratio (RR) of 0.75.

^b^ Candemir et al.^23^ is high‐risk of bias while Chen et al.^24^ and Dharma et al.^26^ are moderate‐risk of bias.

^c^ *I* ^2^ test is more than 80%.

^d^ The 95% CI does not exclude the MD of 0.25.

^e^ The 95% CI does not exclude the null hypothesis (MD of 0).

^f^ The 95% CI does not exclude the null hypothesis (RR of 1).

### 3.4 Primary outcomes

#### 3.4.1 Radial artery spasm (RAS)

**Figure 1:**
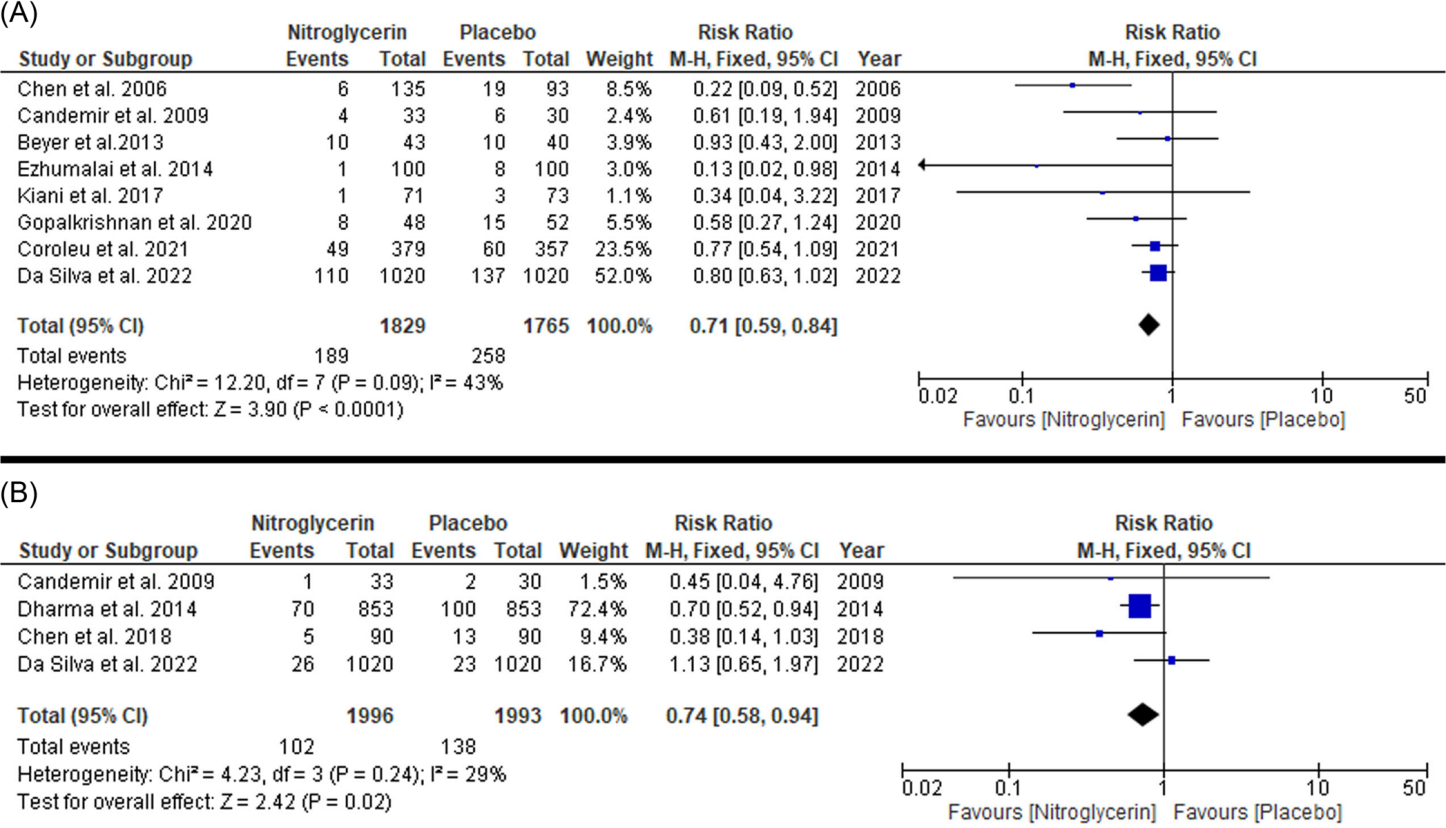
Forest plot of the primary outcomes ((A) RAS and (B) RAO). CI, confidence interval; RAO, radial artery occlusion; RAS, radial artery spasm

The overall pooled risk ratio favored nitroglycerin (regardless of the route of administration) (1829 patients) over placebo (1765 patients) in reducing the incidence of RAS (RR: 0.71 with 95% CI [0.59–0.84], *p* = .0001) (moderate quality evidence). Pooled studies were homogenous (*p* = .09, *I* ^2^ = 43%) (Figure 1A, Table 3). We conducted a subgroup analysis based on the route of administration to evaluate the effect of subcutaneous, topical, and intra‐arterial routes on the RAS incidence; pooled risk ratio favored subcutaneous nitroglycerin (718 patients) over placebo (653 patients) (RR: 0.57 with 95% CI [0.43–0.77], *p* = .0003); however, pooled risk ratio showed no difference between topical nitroglycerin (91 patients) and placebo (92 patients) (RR: 0.73 with 95% CI [0.42–1.24], *p* = .24, neither intra‐arterial nitroglycerin (1020 patients) and placebo (1020 patients) (RR: 0.8 with 95% CI [0.63–1.02], *p* = .07)), respectively (Figure S3).

#### 3.4.2 Radial artery occlusion (RAO)

The overall pooled risk ratio favored nitroglycerin (regardless of the route of administration) (718 patients) over placebo (718 patients) in reducing the incidence of RAO (RR: 0.74 with 95% CI [0.58–0.94], *p* = .02) (low‐quality evidence). Pooled studies were homogenous (*p* = .24, *I* ^2^ = 29%) (Figure 1B, Table 3). We conducted a subgroup analysis based on the route of administration to evaluate the effect of subcutaneous and intra‐arterial routes on the RAS incidence; pooled risk ratio favored subcutaneous nitroglycerin (123 patients) over placebo (120 patients) (RR: 0.39 with 95% CI [0.16–0.98, *p* = .05); however, pooled risk ratio showed no difference between intra‐arterial nitroglycerin (1873 patients) and placebo (1873 patients) (RR: 0.78 with 95% CI [0.6–1.01], *p* = .06) (Figure S4).

### 3.5 Secondary outcomes

#### 3.5.1 Radial artery diameter

**Figure 2:**
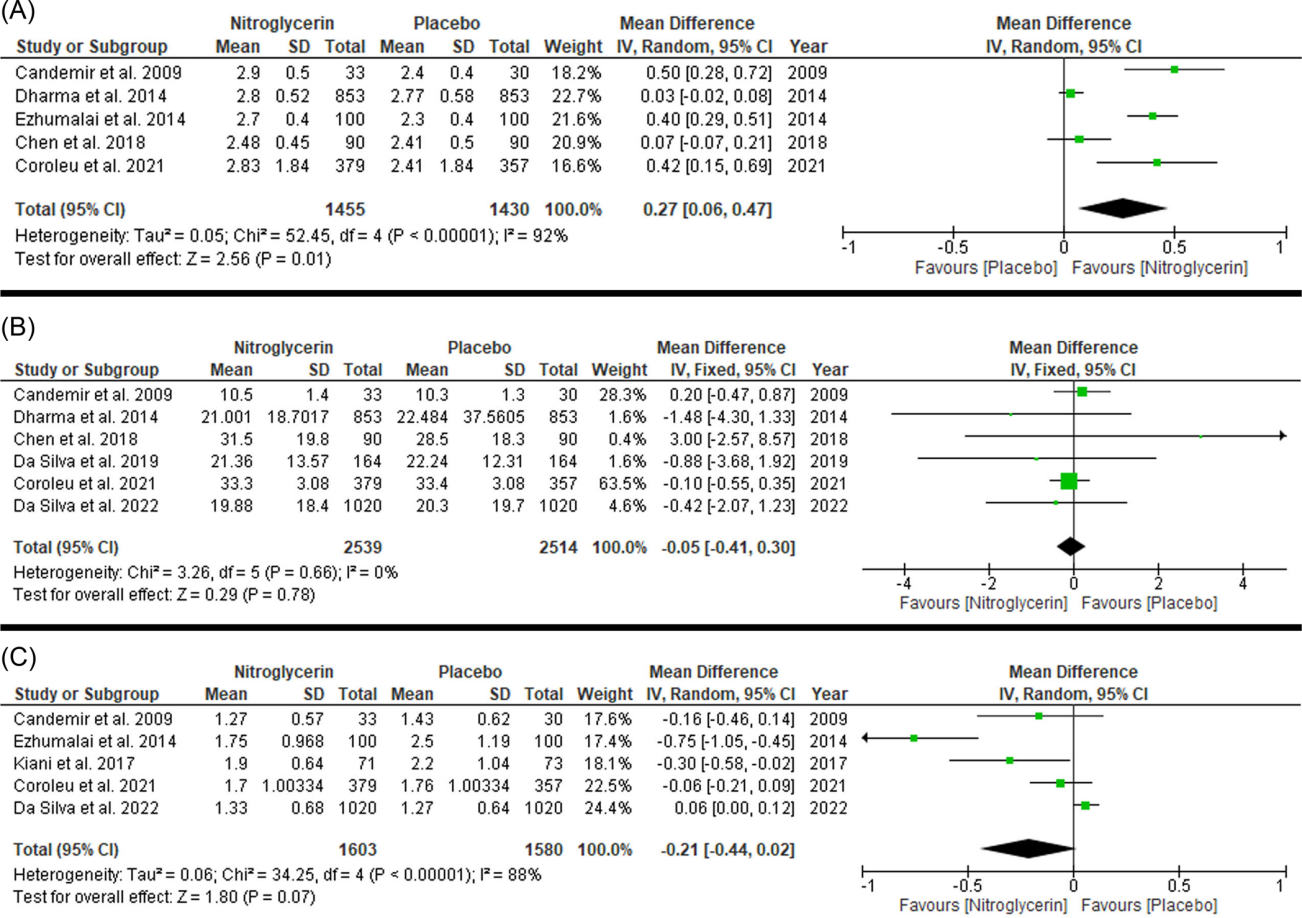
Forest plot of the secondary outcomes ((A) radial artery diameter, (B) procedure duration in minutes, and (C) radial artery puncture attempts)). CI, confidence interval; SD, standard deviation

The overall pooled mean difference ratio favored nitroglycerin (regardless of the route of administration) over placebo in dilating the radial artery (MD: 0.27 with 95% CI [0.06–0.47], *p* = .01) (very low‐quality evidence). Pooled studies were not homogenous (*p* = .00001, *I* ^2^ = 92%) (Figure 2A, Table 3). We conducted a sensitivity analysis by excluding one study in each scenario. However, heterogeneity was not resolved by sensitivity analysis (Table S4). We conducted a subgroup analysis based on the route of administration to evaluate the effect of subcutaneous and intra‐arterial routes on the radial artery diameter; pooled mean difference favored subcutaneous nitroglycerin over placebo (MD: 0.34 with 95% CI [0.13–0.54], *p* = .001); however, pooled mean difference showed no difference between intra‐arterial nitroglycerin and placebo (MD: 0.03 with 95% CI [−0.02 to 0.08], *p* = .26) (Figure S5).

#### 3.5.2 Procedure duration in minutes

The pooled mean difference ratio showed no difference between nitroglycerin (regardless of the route of administration) and placebo (MD: −0.05 with 95% CI [−0.41 to 0.30], *p* = .78) (very low‐quality evidence). Pooled studies were homogenous (*p* = .66, *I* ^2^ = 0%) (Figure 2B, Table 3 **).** We conducted a subgroup analysis based on the route of administration to evaluate the effect of subcutaneous and intra‐arterial routes on the procedure duration; pooled mean difference showed no difference between subcutaneous or intra‐arterial nitroglycerin and placebo ((MD: 0.01 with 95% CI [−0.36 to 0.38], *p* = .98) – (MD: −0.73 with 95% CI [−2.00 to 0.54], *p* = .26)), respectively (Figure S6).

#### 3.5.3 Radial artery puncture attempts

The pooled mean difference ratio showed no difference between nitroglycerin (regardless of the route of administration) and placebo (MD: −0.21 with 95% CI [−0.44 to 0.02], *p* = .07) (very low‐quality evidence). Pooled studies were not homogenous (*p* = .00001, *I* ^2^ = 88%) (Figure 2C, Table 3). We conducted a sensitivity analysis by excluding one study in each scenario. However, heterogeneity was not resolved by sensitivity analysis (Table S4). We conducted a subgroup analysis based on the route of administration to evaluate the effect of subcutaneous and intra‐arterial routes on the radial artery diameter; pooled mean difference favored subcutaneous nitroglycerin over placebo (MD: −0.3 with 95% CI [−0.6 to −0.01], *p* = .04); however, pooled mean difference showed favored placebo over intra‐arterial nitroglycerin (MD: 0.06 with 95% CI [0.00–0.12], *p* = .04) (Figure S7).

#### 3.5.4 Adverse events

The pooled risk ratio showed no difference between nitroglycerin and placebo regarding the incidence of hematoma (RR: 0.69 with 95% CI [0.43–1.10]. *p* = .12) (low‐quality evidence) (Figure S8, Table 3). Pooled studies were homogenous (*p* = .66, *I* ^2^ = 0%). We conducted a subgroup analysis based on the route of administration. The pooled mean difference showed no difference between subcutaneous nor topical nitroglycerin and placebo ((RR: 0.64 with 95% CI [0.39–1.04]. *p* = .07) – (RR: 1.43 with 95% CI [0.30–6.92]. *p* = .65)), respectively (Figure S9).

However, the pooled risk ratio showed that nitroglycerin was associated with more incidence of hypotension (RR: 2.11 with 95% CI [1.20–3.70], *p* = .009) (low‐quality evidence) (Figure S8, Table 3). Pooled studies were homogenous (*p* = .97, *I* ^2^ = 0%). We conducted a subgroup analysis based on the route of administration. The pooled mean difference showed statistically significant result with intra‐arterial nitroglycerin administration (RR: 2.08 with 95% CI [1.05–4.12], *p* = .04). In contrast to subcutaneous nitroglycerin that showed nonsignificant result (RR: 2.16 with 95% CI [0.80–5.83], *p* = .13) (Figure S10).

Finally, the pooled risk ratio showed that nitroglycerin was associated with more incidence of headache (RR: 2.86 with 95% CI [1.25–6.55], *p* = .01) (low‐quality evidence) (Figure S8, Table 3). Pooled studies were heterogeneous (*p* = .09, *I* ^2^ = 54%) We conducted a subgroup analysis based on the route of administration. The pooled mean difference showed statistically significant result with subcutaneous nitroglycerin administration (RR: 5.33 with 95% CI [1.73–16.39], *p* = .004). In contrast to topical nitroglycerin that showed nonsignificant result (RR: 0.54 with 95% CI [0.10–2.82], *p* = .47) (Figure S11).

## 4 DISCUSSION

Our meta‐analysis involving 11 RCTs demonstrated that nitroglycerin is effective in reducing the incidence of RAS and RAO by using subcutaneous nitroglycerin. Moreover, nitroglycerin was effective in increasing the radial artery diameter. However, we found no difference between nitroglycerin and placebo in reducing radial artery puncture attempts and procedure duration.

Regarding RAS incidence (Table S5), subcutaneous nitroglycerin was effective in reducing RAS, while intra‐arterial and topical nitroglycerin were not. RAS can lead to RAO by causing local trauma, flow interruption, and thrombus formation.^36^ RAS also can exacerbate endothelial cell and vascular damage, a mechanism that can also be associated with RAO.^37^ Furthermore, RAS was recently reported to be an independent predictor of RAO.^13^ However, Dhrama et al.^26^ stated that severe RAS was not a predictor of RAO. This difference can be attributed to the variation in the clinical criteria used to detect RAS. Hence, preventing RAS can lead to subsequent prevention of RAO. The variability of the nitroglycerin dose may also affect our findings. For example, regarding the dose of intra‐arterial nitroglycerin: both Dharma et al.^26^ and Da Silva et al.^13^ used 500 μg, Da Silva et al.^21^ used 200 μg, and Chen et al. used 100 μg. On the other hand, the dose of subcutaneous nitroglycerin was 500 μg^17^, ^22^, ^23^, ^24^ except for Coroleu et al.,^25^ who used 200 μg.

Similarly, subcutaneous nitroglycerin was effective in reducing RAO, while intra‐arterial nitroglycerin was not. Three studies (two subcutaneous^23^, ^24^ and one intra‐arterial^26^) favored nitroglycerin, while Da Silva et al.^13^ showed that intra‐arterial nitroglycerin was not effective in reducing RAO. Da Silva et al.^13^ used a hemostasis protocol with early decompression (1–2 h) that can have countered the effect of nitroglycerin. To clarify, nitroglycerin increases the regional flow, subsequently reducing the effect of prolonged compression.^13^ Supporting this hypothesis, hemostasis duration is a predictor of RAO.^26^ Using the same hemostasis protocol, earlier decompression (2 h) is associated with a lower incidence of RAO than later decompression (6 h).^38^

The exact mechanism of RAO remains unclear. An important risk factor for RAO is the development of thrombus following endothelial injury with the reduction in blood flow after sheath and catheter insertion.^39^, ^40^ Furthermore, recurrent radial artery cannulation can cause intimal hyperplasia and increased intima‐media thickness, resulting in detrimental arterial wall remodeling and increased RAO risk.^41^ In addition, blood stasis while achieving hemostasis provides the nidus for thrombus formation, potentially leading to vessel occlusion. Hence, minimizing compression time and using a small introducer sheath can decrease RAO risk by reducing endothelial damage. It is also essential to preserve the long‐term functionality of the radial artery after transradial catheterization to maximize the ultimate benefit. RAO may be painful, but it is usually asymptomatic because of the double blood supply of the hand through the palmar arch; however, it can limit and preclude the use of the radial artery as a future access site.^26^

Despite the small diameter of the radial artery (2.6 ± 0.4 mm), it possesses a thicker tunica media than many other arteries, hence classified as a type III artery graft.^42^, ^43^ Moreover, greater alpha‐1 innervation and stronger endothelin‐1, and angiotensin II receptor‐mediated contractility increase the spasticity of the radial artery.^44^, ^45^ Therefore, the alpha‐1 adrenoceptor dominant nature of the radial artery makes it more prone to spasms provoked by pain and stress; hence, adequate analgesia and sedation can be effective in reducing this effect.^8^ Furthermore, inhibiting sodium channels and blocking action potential at the sympathetic nerves by local anesthetics can potentiate this effect.^46^, ^47^ However, unexpected vasoconstriction has also been documented with lignocaine injection, especially at lower concentrations.^48^ Therefore, the effect of co‐administrated local anesthetics can significantly bias our findings, given the variation between the included studies in the local anesthetic protocol.

Furthermore, the variability in heparin doses may affect the pooled analysis of RAO. The heparin dose was 3000u in Chen et al.^8^ and Chen et al.,^24^ meanwhile both Da Silva et al., Candemir et al., and Coroleu et al. used 5000u.^13^, ^23^, ^25^ Both Dharma et al. and Beyer et al. used weight‐based dosage, 50–100/kg and 50–70 u/kg, respectively.^20^, ^26^ The rest of the included RCTs did not report the heparin dosage.^17^, ^21^, ^22^, ^27^ Unfortunately, we could not perform a subgroup analysis to assess the effect of heparin administration in reducing the RAS, as heparin was an adjuvant drug and was administrated in both the intervention and the control group.

Nitroglycerin's vasodilatory effect is hypothesized to be the mechanism through which it may reduce the rate of RAO.^26^ Moreover, radial artery size influences the accessibility of the radial artery, the ability to insert appropriately sized sheaths, and guide catheters.^20^ In this regard, our meta‐analysis significantly favored nitroglycerin in increasing radial artery diameter. Nitroglycerin causes the radial artery to dilate due to its endothelial relaxing effect through nitric oxide.^49^ Transradial catheterization, radial artery puncture, and mechanical friction with the intima lead to acute inflammatory response, subsequently leading to local edema, and artery contraction.^24^ This response can decrease the luminal diameter of the radial artery for up to 6 months after the procedure.^50^ The nitric oxide donor effect of nitroglycerin counteracts this decrease in endothelial dysfunction, thus preventing subsequent RAO. In addition, nitroglycerin sensitivity is higher in the radial artery than in other arteries.^48^ On the contrary, nitroglycerin was not effective in reducing radial artery puncture attempts and procedure duration. Multiple puncture attempts are a major cause of RAS, which can be a particular problem in catheterization training programs.^51^, ^52^

Regarding the safety of nitroglycerin, on one hand, our analysis showed more incidence of hypotension and headache in the nitroglycerin group compared with the placebo. We can attribute this to the vasodilatory effect of nitroglycerin. Hence, headache and hypotension are more likely to happen after intra‐arterial nitroglycerin because of the high dose and the greater effect on general circulation.^24^, ^53^ In contrast to intra‐arterial nitroglycerin injection, local subcutaneous nitroglycerin injection delivers nitroglycerin to the radial artery more directly and locally, leading to fewer adverse effects.^24^ The same applies to topical nitroglycerin; however, it only works on the radial artery access site; hence, further administration of intra‐arterial nitroglycerin is still required.^20^ All the events were mild to moderate and feasibly managed. On the other hand, our analysis showed no difference in the hematoma incidence between nitroglycerin and placebo. Notably, none of the included trials reported hand ischemia with nitroglycerin nor with placebo. In summary, nitroglycerin is well tolerated and safe for RAS and RAO prevention during transradial catheterization.

## 5 STRENGTHS

To the best of our knowledge, this is the first systematic review and meta‐analysis to discuss the effect of nitroglycerin with the various routes of administration to prevent RAS and RAO during transradial catheterization, hence providing the most subtle evidence in this regard. Moreover, we strictly followed the Preferred Reporting Items for Systematic Reviews and Meta‐Analyses (PRISMA) statement^28^ and the Cochrane Handbook of Systematic reviews and meta‐analysis^29^ and prospectively registered and published our protocol.

## 6 LIMITATIONS

Our study has a few limitations. First, we included two non‐peer‐reviewed trials.^21^, ^27^ Second, the variation in operator experience throughout different trials can lead to significant interobserver and inter‐operator bias affecting our findings. Third, none of the included RCTs discussed if the RAS forced the operator to continue the procedure via other access. To clarify, Kiani et al.^22^ reported that cannulation failed in three patients. Both Chen et al.^24^ and Da Silva et al.^13^ excluded six and 26 patients, respectively due to the radial artery being inaccessible. Furthermore, the variability in the nitroglycerine and heparin doses besides the method of RAS and RAO detection may affect our findings. Fourth, the difference in transradial catheterization techniques, including puncture technique, post‐puncture cocktails, and equipment used, especially sheath size, is reported as a predictor of RAO,^54^ and hemostasis protocols can also affect our findings. Fifth, comorbidities and age‐related atherosclerotic changes in the radial artery are also potential confounding variables that we cannot control. Finally, we detected significant heterogeneity with radial artery diameter and puncture attempts; hence their findings cannot be generalized. Moreover, the GRADE assessment yielded very low‐quality evidence for most of the included outcomes, further limiting the generalizability of our findings.

## 7 IMPLICATIONS FOR FUTURE RESEARCH

Future trials should assess the following: first, the effect of different hemostasis protocols, especially hemostasis duration on nitroglycerin effect in preventing RAO to reach the most proper time for hemostasis compression after transradial catheterization. Second, future trials should depend on the objective assessment of radial artery lumen using duplex ultrasound, which is the gold standard in detecting thrombosis and flow disturbance, rather than the subjective clinical assessment to accurately determine the extent of post‐procedural RAO.^37^ Third, more research is still required to analyze the long‐term patency because spontaneous recanalization is possible for up to 30 days.^26^ Finally, administrating heparin intra‐arterially (via sheath) can lead to arm pain which in turn may increase the risk of RAS; hence, the association between the heparin route of administration and RAS incidence should be addressed in future trials.

## 8 CONCLUSION

Our current review indicates that nitroglycerin is a feasible economic strategy to facilitate transradial catheterization, decreasing the incidence of RAS and subsequent RAO, especially subcutaneous nitroglycerin, while the evidence of nitroglycerin's efficacy in reducing the incidence of RAO, radial artery puncture attempts, and procedure duration is uncertain. More trials are still required to assess the intra‐arterial route's potential efficacy through different puncture techniques, post‐puncture cocktails, equipment, and hemostasis protocols.

## AUTHOR CONTRIBUTIONS

Basel Abdelazeem and Mohamed T. Abuelazm conceived the idea. Basel Abdelazeem designed the research work and plan. Basel Abdelazeem and Mohamed Gamal searched the databases. Sarya Swed and Mohamed Gamal screened the retrieved records, and Mostafa Atef resolved the conflicts. Sarya Swed, Mohamed Gamal, and Mostafa Atef extracted relevant data, assessed the quality of evidence, and resolved the conflicts. Mohamed T. Abuelazm and Basel Abdelazeem performed the analysis. Mohamed T. Abuelazm and Muhammad A. Noori wrote the final manuscript. Annabelle S. Volgman and Anthony Lutz reviewed and edited the manuscript. All authors have read and agreed to the final version of the manuscript.

## CONFLICTS OF INTEREST

The authors declare no conflicts of interest.

## Supporting information

Supporting information.

## Data Availability

Data are available on request.
