# Supplementary material for: The efficacy of nitroglycerin to prevent radial artery spasm and occlusion during and after transradial catheterization: A systematic review and meta‐analysis of randomized controlled trials
Source: Clin Cardiol. 2022 Nov 6;45(12):1171–83. doi: 10.1002/clc.23906 (PMC9748765; doi:10.1002/clc.23906)
Supplement: Supplementary file 1 — Supporting information. [file CLC-45-1171-s001.docx]

**Title.**

**The Efficacy of Nitroglycerine to Prevent Radial Artery Spasm and Occlusion during and after Trans-Radial Catheterization: A Systematic Review and Meta-Analysis of Randomized Controlled Trials.**

**Running Title.**

Nitroglycerine to Prevent Radial Artery Spasm or Occlusion

**Authors.**

Basel Abdelazeem MD^1,2*^, Mohamed T. Abuelazm MBBCh Candidate^3*^, Sarya Swed MBBCh Candidate^4^, Mohamed Gamal MBBCh Candidate^3^, Mostafa Atef MBBCh Candidate ^5^, Mohamed A. Al-Zeftawy MBBCh Candidate^3^, Muhammad A. Noori MD^6^, Anthony Lutz DO, FACC, FACOI^2,7^, Annabelle S.Volgman MD, FACC, FAHA^8^.
*, Both authors have equal contribution.

**Affiliations.**

1. McLaren Health Care, Flint, Michigan, USA.
2. Michigan State University, East Lansing, Michigan, USA.
3. Faculty of Medicine, Tanta University, Tanta, Egypt.
4. Faculty of Medicine, Aleppo University, Aleppo, Syria.
5. Faculty of Medicine, Cairo University, Cairo, Egypt
6. Rutgers Health/Trinitas, New Jersey, USA.
7. Division of Cardiology, Beaumont Hospital, Farmington Hills, Michigan, USA.
8. Division of Cardiology, Rush University Medical Center, Chicago, IL, USA

**Keywords.**

Nitroglycerin; RAS; RAO; Trans-radial; cardiac intervention; radial artery occlusion; radial artery spasm

**Corresponding author.**

Sarya Swed, MBBCh Candidate

Address: Aleppo, Alshahba.

Tel: +963-936500813.

Email: saryaswed1@gmail.com.

ORCID: 0000-0002-9983-2020.

**Contents:**

**Tables.**

Table S1: PRISMA 2020 checklist.

Table S2: Search terms and results in different databases.

Table S3: Author judgment for ROB assessment.

Table S4: Sensitivity analysis.

Table S5: incidence of RAS & RAO.

**Figures.**

Figure S1: PRISMA flow chart of the screening process.

Figure S2: Summary of risk of bias (A- review authors' judgments about each risk of bias item for each included study, B- review authors' judgments about each risk of bias item presented as percentages across all included studies).

Figure S3: Forest plot of Subgroup analysis, outcome: Radial artery spasm.

Figure S4: Forest plot of Subgroup analysis, outcome: Radial artery occlusion.

Figure S5: Forest plot of Subgroup analysis, outcome: Radial artery diameter.

Figure S6: Forest plot of Subgroup analysis, outcome: Procedure duration in minutes.

Figure S7: Forest plot of Subgroup analysis, outcome: Radial artery puncture attempts.

Figure S8: Forest plot of the adverse events.

Figure S9: Forest plot of hematoma adverse effect and subgroup analysis.

Figure S10: Forest plot of hypotension adverse effect and subgroup analysis.

Figure S11: Forest plot of headache adverse effect and subgroup analysis.

| **Section and Topic** | **Item #** | **Checklist item** | **Location where item is reported** |
| --- | --- | --- | --- |
| **TITLE** | | |  |
| Title | 1 | Identify the report as a systematic review. | Line 2 |
| **ABSTRACT** | | |  |
| Abstract | 2 | See the PRISMA 2020 for Abstracts checklist. | Page 3 |
| **INTRODUCTION** | | |  |
| Rationale | 3 | Describe the rationale for the review in the context of existing knowledge. | Page 5 |
| Objectives | 4 | Provide an explicit statement of the objective(s) or question(s) the review addresses. | Page 6 |
| **METHODS** | | |  |
| Eligibility criteria | 5 | Specify the inclusion and exclusion criteria for the review and how studies were grouped for the syntheses. | Page 7, subsection 2.3 |
| Information sources | 6 | Specify all databases, registers, websites, organisations, reference lists and other sources searched or consulted to identify studies. Specify the date when each source was last searched or consulted. | Page 7, subsection 2.2 |
| Search strategy | 7 | Present the full search strategies for all databases, registers and websites, including any filters and limits used. | Supplementary material, table S2 |
| Selection process | 8 | Specify the methods used to decide whether a study met the inclusion criteria of the review, including how many reviewers screened each record and each report retrieved, whether they worked independently, and if applicable, details of automation tools used in the process. | Page 8, subsection 2.4 |
| Data collection process | 9 | Specify the methods used to collect data from reports, including how many reviewers collected data from each report, whether they worked independently, any processes for obtaining or confirming data from study investigators, and if applicable, details of automation tools used in the process. | Page 8, subsection 2.5 |
| Data items | 10a | List and define all outcomes for which data were sought. Specify whether all results that were compatible with each outcome domain in each study were sought (e.g. for all measures, time points, analyses), and if not, the methods used to decide which results to collect. | Page 8, subsection 2.5 |
|  | 10b | List and define all other variables for which data were sought (e.g. participant and intervention characteristics, funding sources). Describe any assumptions made about any missing or unclear information. | Page 8, subsection 2.5 |
| Study risk of bias assessment | 11 | Specify the methods used to assess risk of bias in the included studies, including details of the tool(s) used, how many reviewers assessed each study and whether they worked independently, and if applicable, details of automation tools used in the process. | Page 8, subsection 2.6 |
| Effect measures | 12 | Specify for each outcome the effect measure(s) (e.g. risk ratio, mean difference) used in the synthesis or presentation of results. | Page 9, subsection 2.7 |
| Synthesis methods | 13a | Describe the processes used to decide which studies were eligible for each synthesis (e.g. tabulating the study intervention characteristics and comparing against the planned groups for each synthesis (item #5)). | Page 9, subsection 2.7 |
|  | 13b | Describe any methods required to prepare the data for presentation or synthesis, such as handling of missing summary statistics, or data conversions. | Page 9, subsection 2.7 |
|  | 13c | Describe any methods used to tabulate or visually display results of individual studies and syntheses. | Page 9, subsection 2.7 |
|  | 13d | Describe any methods used to synthesize results and provide a rationale for the choice(s). If meta-analysis was performed, describe the model(s), method(s) to identify the presence and extent of statistical heterogeneity, and software package(s) used. | Page 9, subsection 2.7 |
|  | 13e | Describe any methods used to explore possible causes of heterogeneity among study results (e.g. subgroup analysis, meta-regression). | Page 9, subsection 2.7 |
|  | 13f | Describe any sensitivity analyses conducted to assess robustness of the synthesized results. | Page 9, subsection 2.7 |
| Reporting bias assessment | 14 | Describe any methods used to assess risk of bias due to missing results in a synthesis (arising from reporting biases). | Page 8, subsection 2.6 |
| Certainty assessment | 15 | Describe any methods used to assess certainty (or confidence) in the body of evidence for an outcome. | Page 8, subsection 2.6 |
| **RESULTS** | | |  |
| Study selection | 16a | Describe the results of the search and selection process, from the number of records identified in the search to the number of studies included in the review, ideally using a flow diagram. | Page 10, subsection 3.1 |
|  | 16b | Cite studies that might appear to meet the inclusion criteria, but which were excluded, and explain why they were excluded. | Not applicable |
| Study characteristics | 17 | Cite each included study and present its characteristics. | Page 10, subsection 3.2 |
| Risk of bias in studies | 18 | Present assessments of risk of bias for each included study. | Page 10, subsection 3.3 |
| Results of individual studies | 19 | For all outcomes, present, for each study: (a) summary statistics for each group (where appropriate) and (b) an effect estimate and its precision (e.g. confidence/credible interval), ideally using structured tables or plots. | Pages 11-12, subsections 3.4-3.5 |
| Results of syntheses | 20a | For each synthesis, briefly summarise the characteristics and risk of bias among contributing studies. | Page 10, subsection 3.3 |
|  | 20b | Present results of all statistical syntheses conducted. If meta-analysis was done, present for each the summary estimate and its precision (e.g. confidence/credible interval) and measures of statistical heterogeneity. If comparing groups, describe the direction of the effect. | Pages 11-13, subsections 3.4-3.5 |
|  | 20c | Present results of all investigations of possible causes of heterogeneity among study results. | Pages 11-12, subsections 3.4-3.5 |
|  | 20d | Present results of all sensitivity analyses conducted to assess the robustness of the synthesized results. | Not applicable |
| Reporting biases | 21 | Present assessments of risk of bias due to missing results (arising from reporting biases) for each synthesis assessed. | Page 10, subsection 3.3 |
| Certainty of evidence | 22 | Present assessments of certainty (or confidence) in the body of evidence for each outcome assessed. | Table 3 |
| **DISCUSSION** | | |  |
| Discussion | 23a | Provide a general interpretation of the results in the context of other evidence. | Page 13 |
|  | 23b | Discuss any limitations of the evidence included in the review. | Page 18 |
|  | 23c | Discuss any limitations of the review processes used. | Page 18 |
|  | 23d | Discuss implications of the results for practice, policy, and future research. | Page 18,19 |
| **OTHER INFORMATION** | | |  |
| Registration and protocol | 24a | Provide registration information for the review, including register name and registration number, or state that the review was not registered. | Page 7, subsection 2.1 |
|  | 24b | Indicate where the review protocol can be accessed, or state that a protocol was not prepared. | Page 7, subsection 2.1 |
|  | 24c | Describe and explain any amendments to information provided at registration or in the protocol. | Page 7, subsection 2.1 |
| Support | 25 | Describe sources of financial or non-financial support for the review, and the role of the funders or sponsors in the review. | Page 2 |
| Competing interests | 26 | Declare any competing interests of review authors. | Page 2 |
| Availability of data, code and other materials | 27 | Report which of the following are publicly available and where they can be found: template data collection forms; data extracted from included studies; data used for all analyses; analytic code; any other materials used in the review. | Page 2 |

Table S1 PRISMA 2020 checklist

| Database | Search Terms | Search Field | Search Results |
| --- | --- | --- | --- |
| Pubmed | (("Nitroglycerin"[Mesh] OR Glyceryl Trinitrate OR Trinitrate, Glyceryl OR Nitrolan OR Nitrospan OR Nitrostat OR Perlinganit OR Nitrong OR Susadrin OR Sustac OR Sustak OR Sustonit OR Transderm Nitro OR Nitroglyn OR Trinitrin OR Trinitrolong OR Anginine OR Dynamite OR Gilustenon OR Nitrangin OR Nitro-Bid OR Nitro Bid OR NitroBi OR Nitro-Dur OR Nitro Dur OR NitroDur OR Nitrocard OR Nitroderm OR Nitroderm TTS OR Nitrol OR Tridil OR Agents, Vasodilator OR Vasodilators OR Vasorelaxant OR Vasodilator OR Vasodilator Agent OR Agent, Vasodilator OR Vasodilator Drug OR Drug, Vasodilator OR Vasodilator Drugs OR Drugs, Vasodilator OR Vasorelaxants OR Vasoactive Antagonists OR Antagonists, Vasoactive) AND ("Radial Artery"[Mesh] OR "radial artery occlusion" OR "radial artery spasm" OR "transradial")) | All Field | 609 |
| Cochrane | (Nitroglycerin) OR (Glyceryl Trinitrate) OR (Nitrolan) OR (Nitrospan) OR (Nitrostat) OR (Perlinganit) OR (Nitrong) OR (Susadrin) OR (Sustac) OR (Sustak) OR (Sustonit) OR (Transderm Nitro) OR (Nitroglyn) OR (Trinitrin) OR (Trinitrolong) OR (Anginine) OR (Dynamite) OR (Gilustenon) OR (Nitrangin) OR (Nitro-Bid) OR (NitroBi) OR (Nitro-Dur) OR (Nitrocard) OR (Nitroderm) OR (Nitroderm TTS) OR (Nitrol) OR (Tridil) OR (Vasodilators) OR (Vasodilator Agent) OR (Vasorelaxants) OR (Vasoactive Antagonists) 6155  (Radial Artery) OR (radial artery occlusion) OR (radial artery spasm) OR (transradial) 2979  1 AND 2 | All Field | 195 |
| WOS | (Nitroglycerin OR Agents, Vasodilator OR Vasodilators OR Vasorelaxant OR Vasodilator OR Vasodilator Agent OR Agent, Vasodilator OR Vasodilator Drug OR Drug, Vasodilator OR Vasodilator Drugs OR Drugs, Vasodilator OR Vasorelaxants OR Vasoactive Antagonists OR Antagonists, Vasoactive) AND (radial artery OR radial artery occlusion OR radial artery spasm OR transradial) | All Field | 438 |
| SCOPUS | (Nitroglycerin OR Agents, Vasodilator OR Vasodilators OR Vasorelaxant OR Vasodilator OR Vasodilator Agent OR Agent, Vasodilator OR Vasodilator Drug OR Drug, Vasodilator OR Vasodilator Drugs OR Drugs, Vasodilator OR Vasorelaxants OR Vasoactive Antagonists OR Antagonists, Vasoactive) AND (radial artery OR radial artery occlusion OR radial artery spasm OR transradial) | Title, Abstract, Keywords | 47 |
| EMBASE | #3. #1 AND #2 1,205 23 Apr 2022  #2. 'radial artery'/exp OR 'radial artery' OR (radial 29,157 23 Apr 2022  AND ('artery'/exp OR artery)) OR (radial AND  artery AND occlusion) OR (radial AND artery AND  spasm) OR transradial  #1. 'nitroglycerin'/exp OR nitroglycerin OR (glyceryl 90,100 23 Apr 2022  AND trinitrate) OR nitrolan OR nitrospan OR  nitrostat OR perlinganit OR nitrong OR susadrin  OR sustac OR sustak OR sustonit OR (transderm AND  nitro) OR nitroglyn OR trinitrin OR trinitrolong  OR anginine OR dynamite OR gilustenon OR  nitrangin OR 'nitro bid' OR (nitro AND bid) OR  nitrobi OR 'nitro dur' OR nitrodur OR nitrocard  OR 'glyceryl trinitrate' OR nitrol OR tridil OR  'vasodilator agent' OR vasodilators OR  vasorelaxant OR (vasodilator AND drugs) OR  (vasoactive AND antagonists) OR vasorelaxants | All Field | 1205 |
| Google Scholar | (Nitroglycerin) AND (radial artery OR radial artery occlusion OR radial artery spasm OR transradial) | All Field | 200 |

Table S2. Search terms and results in different databases.

| Author, Year | Domain | Author Judgment |
| --- | --- | --- |
| Bayer et al. 2013 | Other bias | Unclear- unclear experience of the operator |
| Candemir et al. 2009 | Random sequence generation (selection bias) | High- unrandomized clinical trial |
|  | Allocation concealment (selection bias) | High- unrandomized clinical trial |
|  | Selective reporting (reporting bias) | Unclear- no protocol |
|  | Other bias | High- operator bias based on experience with the trans-radial approach |
| Chen et al. 2006 | Random sequence generation (selection bias) | Unclear- No mention of the method |
|  | Allocation concealment (selection bias) | Unclear- No mention of the method |
|  | Selective reporting (reporting bias) | Unclear- no protocol |
| Chen et al. 2018 | Random sequence generation (selection bias) | Unclear- No mention of the method |
|  | Allocation concealment (selection bias) | Unclear- No mention of the method |
|  | Blinding of participants and personnel (performance bias) | High- Single blinded |
| Dharma et al. 2014 | Selective reporting (reporting bias) | Unclear- no protocol |
|  | Other bias | High- differences in puncture technique, post puncture cocktails, equipment used, and institutional hemostasis protocols |

Table S3 Author judgment for ROB assessment.

| Outcome | No. of  participants (Nitroglycerin/placebo) | No. of  trials | Quantitative data synthesis | | | | Heterogeneity analysis | | |
| --- | --- | --- | --- | --- | --- | --- | --- | --- | --- |
|  |  |  | MD | 95% CI | Z value | p-value | df | p-value | I2 (%) |
| **Radial artery diameter** | | | | | | | | | |
| All studies | 1455/1430 | 5 | 0.27 | [0.06, 0.47] | 2.56 | 0.01 | 4 | 0.00001 | 92 |
| Omitting Candemir et al. 2009 | 1422/1400 | 4 | 0.22 | [0.00,0.43] | 1.97 | 0.05 | 3 | 0.00001 | 93 |
| Omitting El Dharma et al. 2014 | 602/577 | 4 | 0.34 | [0.13,0.54] | 3.21 | 0.001 | 3 | 0.0006 | 83 |
| Omitting Ezhumalai et al. 2014 | 1355/1330 | 4 | 0.23 [0.02, 0.43] | [0.02, 0.43] | 2.16 | 0.03 | 3 | 0.0001 | 87 |
| Omitting Chen et al. 2018 | 1365/1340 | 4 | 0.33 | [0.06, 0.60] | 2.36 | 0.02 | 3 | 0.00001 | 94 |
| Omitting Coroleu et al. 2021 | 1076/1073 | 4 | 0.24 | [0.01, 0.46] | 2.08 | 0.04 | 3 | 0.00001 | 94 |
| **Radial artery puncture attempts** | | | | | | | | | |
| All studies | 1603/1580 | 5 | -0.21 | [-0.44, 0.02] | 1.8 | 0.07 | 4 | 0.00001 | 88 |
| Omitting  Candemir et al. 2009 | 1570/1550 | 4 | -0.23 | [-0.50, 0.04] | 1.66 | 0.1 | 3 | 0.00001 | 91 |
| Omitting El Ezhumalai et al. 2014 | 1503/1480 | 4 | -0.07 | [-0.21, 0.08] | 0.91 | 0.36 | 3 | 0.02 | 68 |
| Omitting Kiani et al. 2017 | 1532/1507 | 4 | -0.19 | [-0.45, 0.06] | 1.47 | 0.14 | 3 | 0.00001 | 90 |
| Omitting Coroleu et al. 2021 | 1224/1223 | 4 | -0.27 | [-0.63, 0.09] | 1.47 | 0.14 | 3 | 0.00001 | 91 |
| Omitting Da Silva et al. 2022 | 583/560 | 4 | -0.30 | [-0.60, -0.01] | 2.02 | 0.04 | 3 | 0.0007 | 82 |

Table S4 Sensitivity analysis

CI: confidence interval; df: degrees of freedom; MD: mean difference

| Study ID | Incidence of RAS N. (%) | | Incidence of RAO N. (%) | |
| --- | --- | --- | --- | --- |
|  | Nitroglycerine | Control | Nitroglycerine | Control |
| Chen et al. 2006 | 6 (4.44) | 19 (20.43) | N/A | N/A |
| Dharma et al. 2014 | N/A | N/A | 70 (8.2) | 100 (11.72) |
| Da Silva et al. 2022 | 110 (0.98) | 137 (13.43) | 26 (2.54) | 23 (2.25) |
| Candemir et al. 2009 | 4 (12.12) | 6 (20) | 1 (3.03) | 2 (6.66) |
| Ezhumalai et al. 2014 | 1 (1) | 8 (8) | N/A | N/A |
| Kiani et al. 2017 | 1 (1.41) | 3 (4.1) | N/A | N/A |
| Chen et al. 2018 | N/A | N/A | 5 (5.31) | 13 (13.82) |
| Coroleu et al. 2021 | 49 (12.9) | 60 (16.8) | N/A | N/A |
| Beyer et al.2013 | 11 (25.58) | 10 (25) | N/A | N/A |
| Gopalkrishnan et al. 2020 | 8 (16.66) | 15 (28.84) | N/A | N/A |

Table S5 incidence of RAS & RAO

N/A: not available; RAS: radia artery spasm; RAO: radial artery occlusion; N: number.


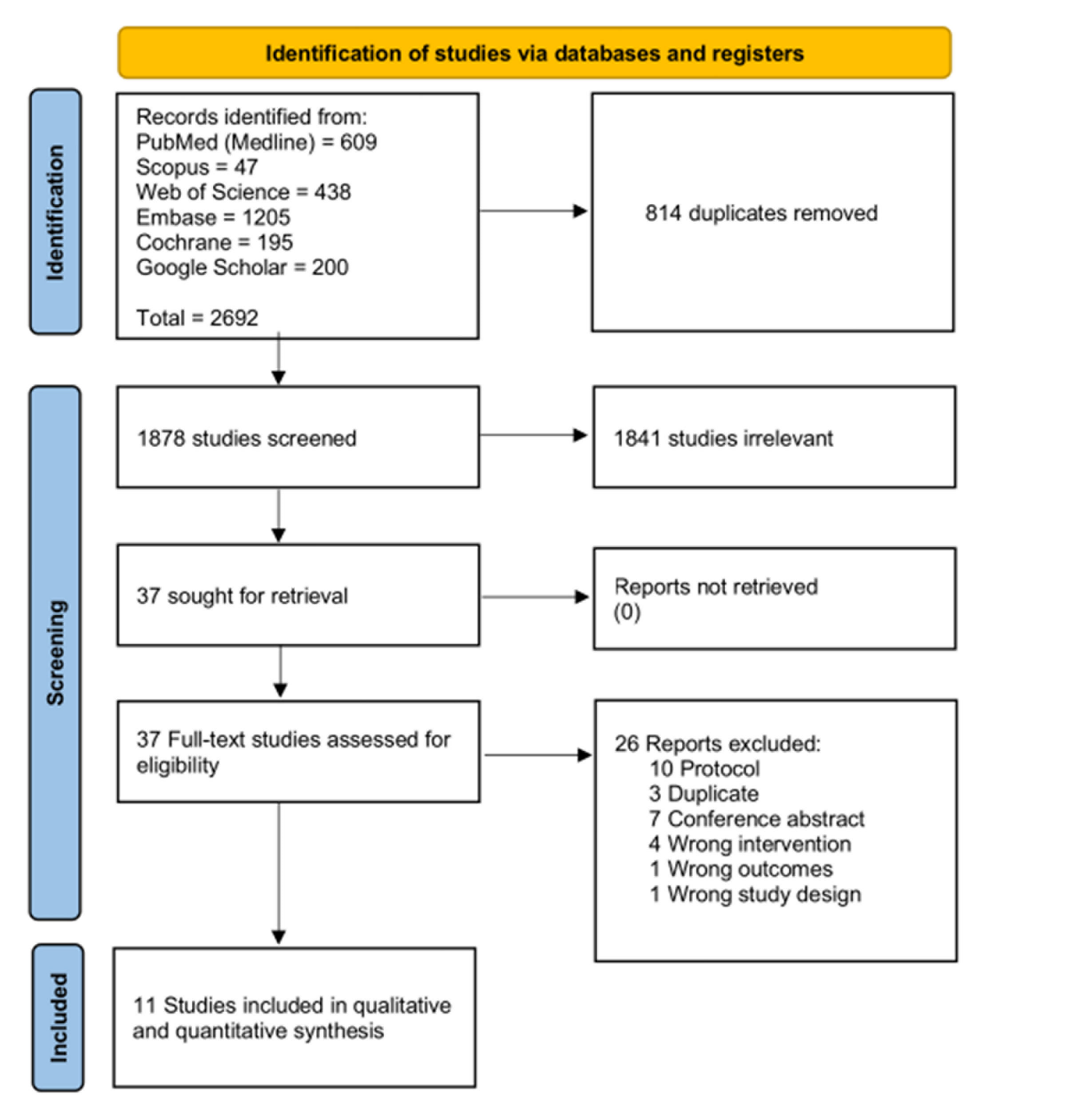
Figure S1: PRISMA flow chart of the screening process.


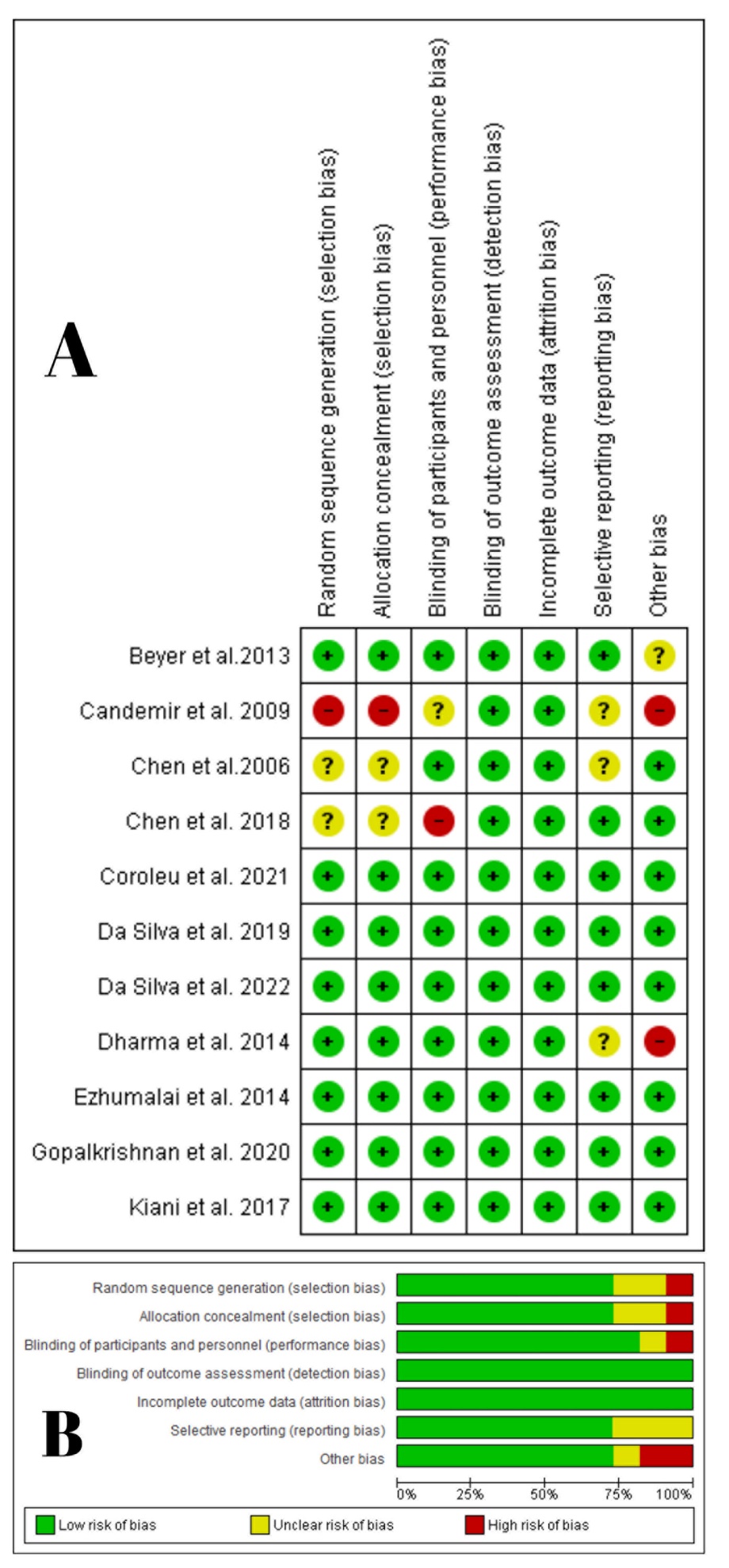


Figure S2: Summary of risk of bias (A- review authors' judgments about each risk of bias item for each included study, B- review authors' judgments about each risk of bias item presented as percentages across all included studies).


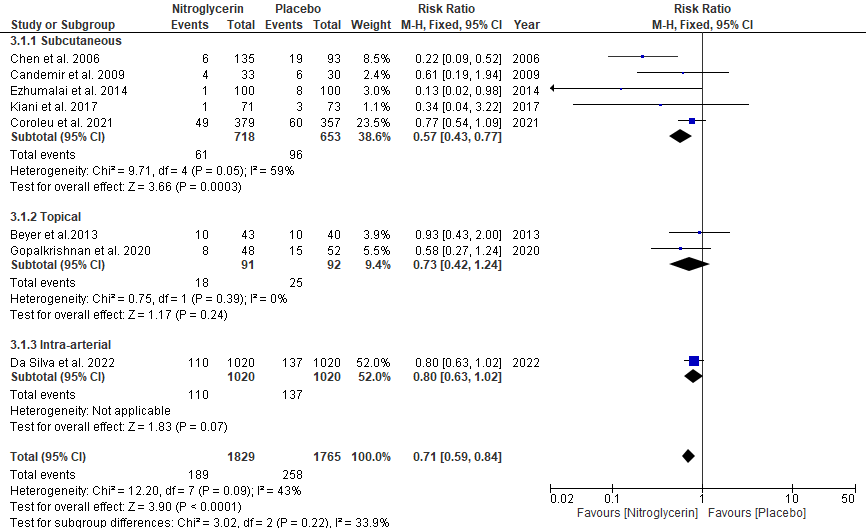


Figure S3 Forest plot of: Subgroup analysis, outcome: Radial artery spasm.


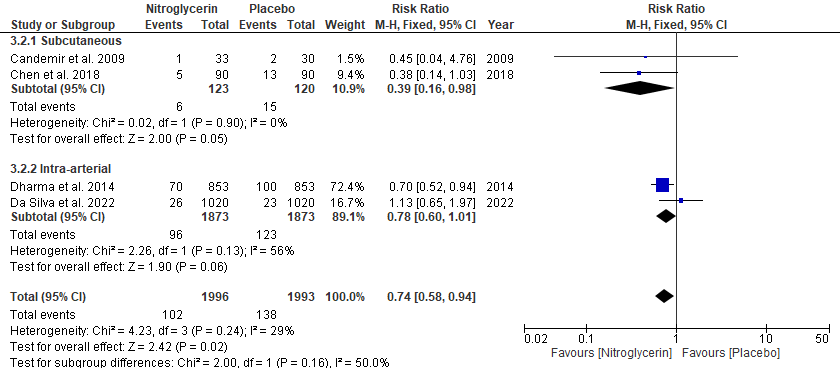


Figure S4: Forest plot of: Subgroup analysis, outcome: Radial artery occlusion.


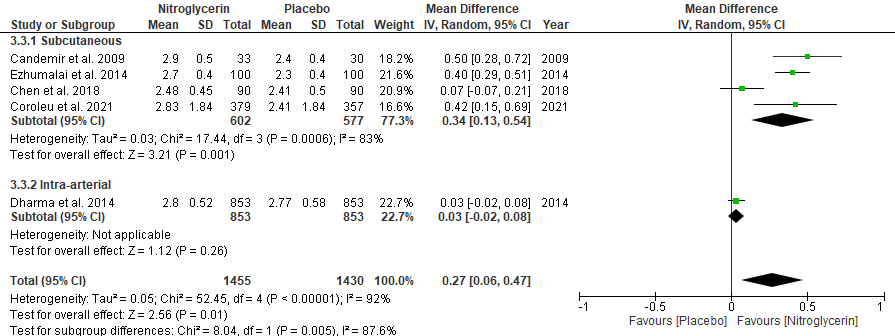


Figure S5 Forest plot of: Subgroup analysis, outcome: Radial artery diameter.

*
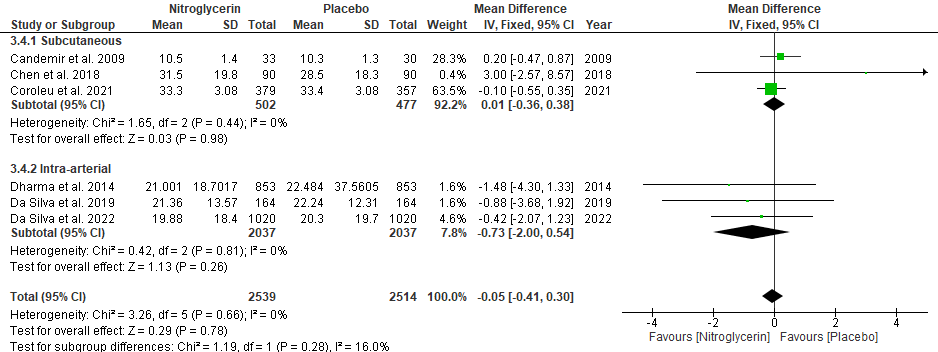
*

Figure S6 Forest plot of: Subgroup analysis, outcome: Procedure duration in minutes.


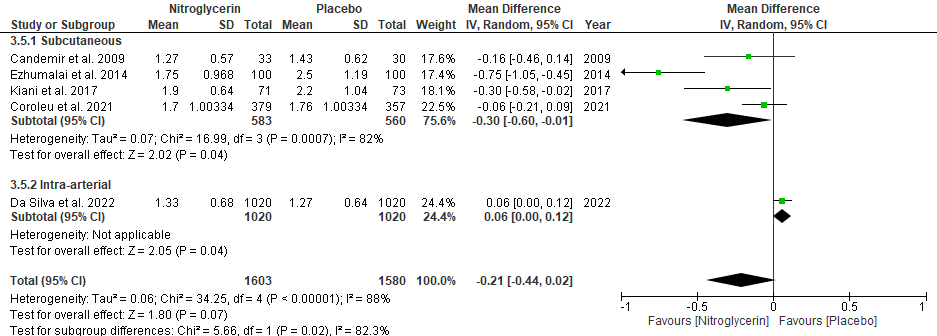


Figure S7 Forest plot of: Subgroup analysis, outcome: Radial artery puncture attempts.


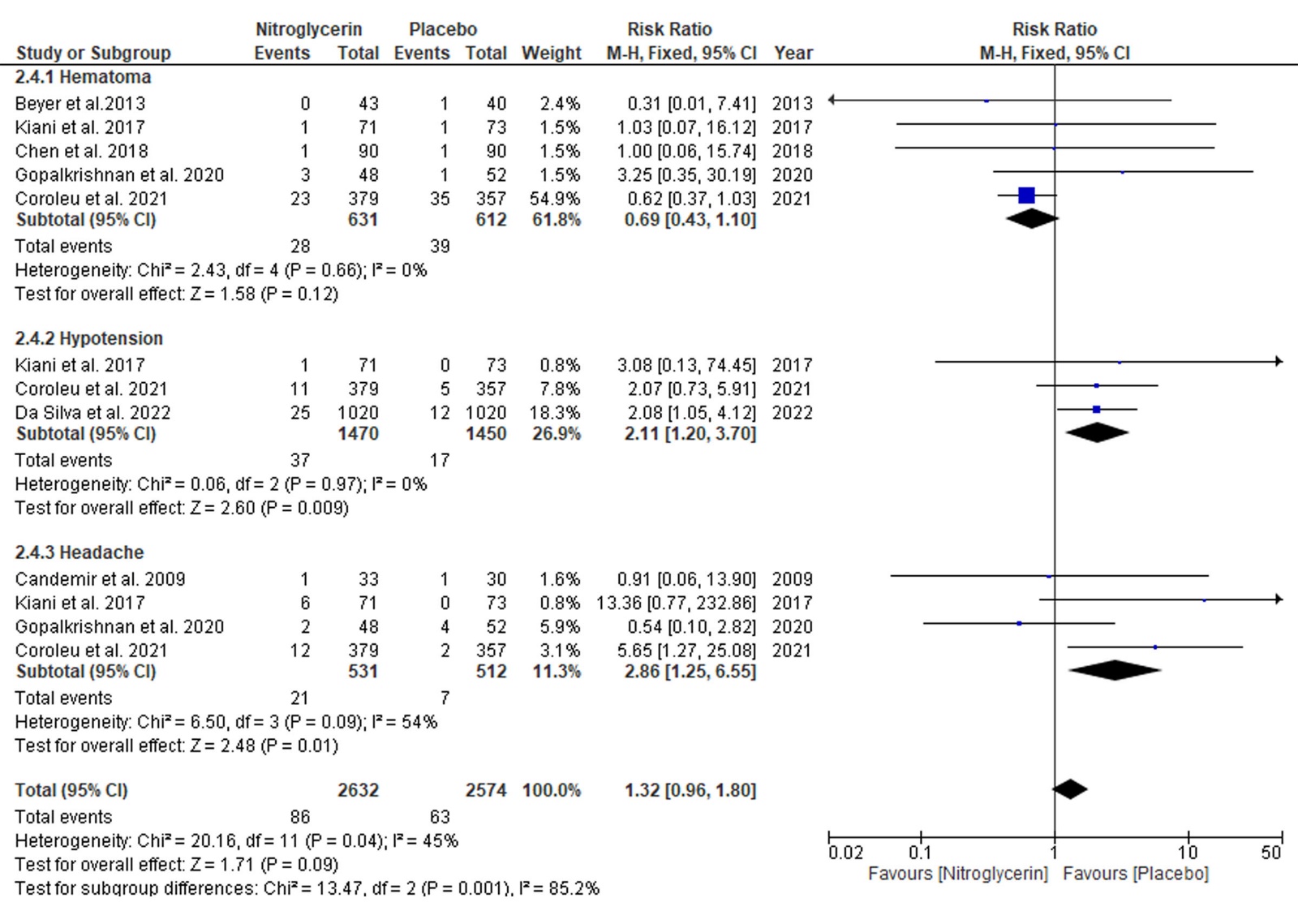


Figure S8 Forest plot of the adverse events.


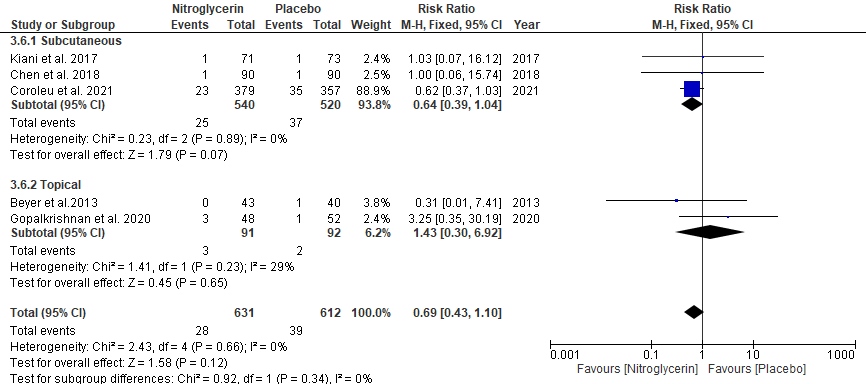


Figure S9 Forest plot of hematoma adverse effect and subgroup analysis


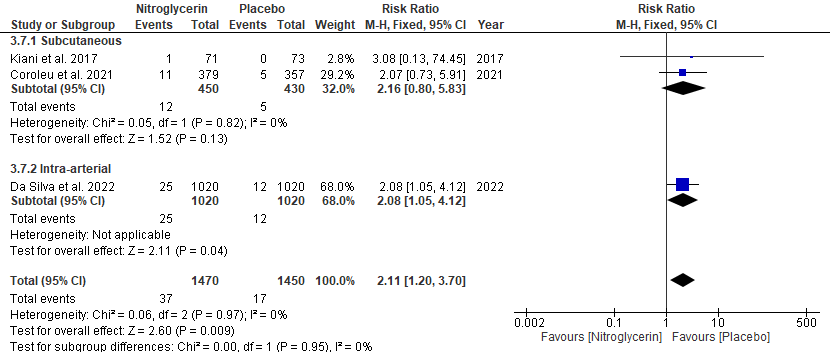


Figure S10 Forest plot of hypotension adverse effect and subgroup analysis


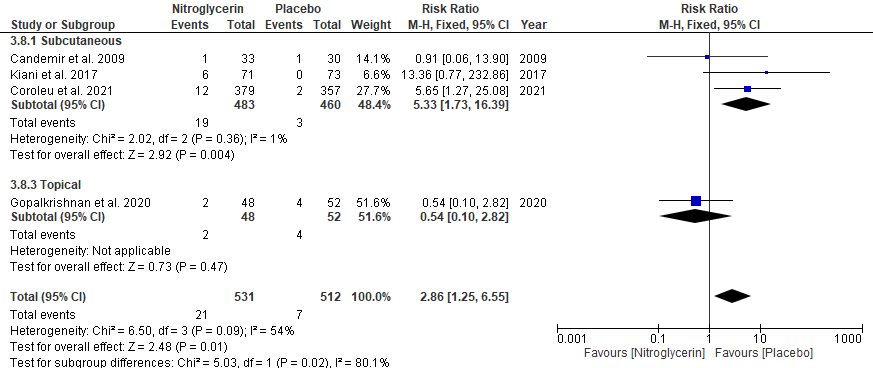


Figure S11 Forest plot of headache adverse effect and subgroup analysis
